# Supplementary material for: Fabrication of Polyelectrolyte Membranes of Pectin Graft-Copolymers with PVA and Their Composites with Phosphomolybdic Acid for Drug Delivery, Toxic Metal Ion Removal, and Fuel Cell Applications
Source: Membranes (Basel). 2021 Oct 18;11(10):792. doi: 10.3390/membranes11100792 (PMC8537921; doi:10.3390/membranes11100792)
Supplement: Supplementary file 1 [file membranes-11-00792-s001.zip › membranes-1408061-supplementary.pdf]

Fabrication of Polyelectrolyte Membranes of Pectin Graft-copolymers with PVA and their Composites with Phosphomolybdic Acid for Drug Delivery, Toxic Metal Ion Removal and Fuel Cell Applications

Raagala Vijitha [1], N. Sivagangi Reddy [1], K. Nagaraja [1], T.J. Sudha Vani [1], Marlia M. Hanafiah[2,3], Katta Venkateswarlu[4], Sivarama Krishna Lakkaboyana[5], K.S.V. Krishna Rao [1,*] and Kummara Madhususdana Rao [6,*]

[1] Polymer Biomaterial Design and Synthesis Laboratory, Department of Chemistry, Yogi Vemana University, Kadapa 516005, Andhra Pradesh, India; vijitha.ragaala@gmail.com (R.V.); siva0406@gmail.com (N.S.R); nagarajakasula33@gmail.com (K.N.); sudhatiruchuru@gmail.com (T.J.S.)

[2] Department of Earth Sciences and Environment, Faculty of Science and Technology, Universiti Kebangsaan Malaysia, Bangi 43600, Selangor, Malaysia; mhmarlia@ukm.edu.my

[3] Centre for Tropical Climate Change System, Institute of Climate Change, Universiti, Kebangsaan Malaysia, Bangi 43600, Selangor, Malaysia

[4] Laboratory for Synthetic & Natural Products Chemistry, Department of Chemistry, Yogi Vemana University, Kadapa 516005, Andhra Pradesh, India; kvenkat@yogivemanauniversity.ac.in

[5] Department of Chemical Technology, Chulalongkorn University, Pathumwan, Bangkok 10330, Thailand; svurams@gmail.com

[6] School of Chemical Engineering, Yeungnam University, 280 Daehak-Ro, Gyeongsan, Gyeongbuk, 38541, South of Korea; msraochem@yu.ac.kr

* Correspondence: ksvkr@yogivemanauniversity.ac.in or drksvkrishna@yvu.edu.in (K.S.V.K.R); msraochem@yu.ac.kr (K.M.R.); Tel.: +91-9704278890 (K.S.V.K.R)

Synthesis of Pectin graft copolymers with (2-acrylado-2-methyl-1-propanesulphonic acid) and (sodium 4-vinylbenzenesulfonate)

Pectin graft copolymers were synthesised as per the our previous work [1]. Briefly, about 0.5g of pectin powder was dispersed in 50 ml of DD water contained three necked round bottomed flask and allowed for overnight stirring at 100 rpm to obtain a clear solution pectin. The flask was fitted with the reflux condenser with nitrogen line and placed the flask into the water bath at 65 ^{o}C. Initiator, potassium persulphate, was dissolved in 5 mL of DD water separately, added to dropwise to the pectin solution and allowed for 30 minutes to generate free radicals. Then required quantities of AMPS and SVBS was mixed with 10 ml of water was added to the pectin solution and stirred for 2h. After completion of rection, reaction mixture cooled to room temperature under tap water, the resulting solution was added to acetone drop wise to get precipitated the graft copolymer. Finally, the graft copolymers (PC-g-AMPS & PC-g-SVBS) were purified as per the Scheme 1 and dried at 40 ^{o}C for 48 h and kept in dedicator for further application.

FTIR Studies

The structural modification of PC-g-AMPS and PC-g-SVBS were confirmed by FTIR spectroscopic studies [2,3]. Figure S1 to comprises the FTIR spectra of pectin, PC-g-AMPS and PC-g-SVBS graft copolymers. From this spectrum of pure pectin, the absorption peaks around 3391, 2941, 1745 & 1631 cm^{-1} corresponds to O-H, aliphatic C-H, carboxylic C=O stretching. The peaks at 1408 & 1345 cm^{-1} is assigned to CH_2 scissoring and O-H bending. A peak at 1069 cm^{-1} corresponds to CH-O-CH stretching. FTIR spectrum of AMPS grafted pectin shows significant peaks at 3100-3600 cm^{-1} corresponds to O-H and N-H stretching. Peaks at 1745 and 1649 cm^{-1} responsible for -C=O stretching vibrations of carboxylic and amide groups. In addition, peaks at 1554 cm^{-1} and 2927 cm^{-1} are responsible for N-H bending and aliphatic C-H stretching vibrations, respectively. The two peaks at 1445 and 1370 cm^{-1} are assigned to CH_2 scissoring and O-H bending responsible for S=O. FTIR spectrum of SVBS grafted pectin shows peaks at 3439, 2924 and 2824 cm^{-1} are responsible for O-H and C-H stretching vibrations, respectively; in addition, 1457 and 1377 cm-1 are responsible for O-H and C-H bending vibrations. The two peaks 1412 and 1127 cm^{-1} are responsible for S=O group. In addition to the pectin peaks, additional peaks appearing in the graft copolymers i.e., PC-g-AMPS and PC-g-SVBS are indicate that the grafting reaction is confirmed (Scheme S1).

NMR studies of graft copolymers

In the ^{1}H NMR spectrum (Figure S2) of PC-g-AMPS the peaks at δ = 6.14-5.98 ppm, 5.56 ppm, and 3.99-2.93 ppm correspond to the pectin back-bone and the peaks at δ = 2.09 ppm and 1.38 ppm corresponds to the AMPS portion.

In the ^{1}H NMR (D_2O) spectrum of PC-g-SVBS, a peak at δ = 7.42 ppm is corresponds to SVBS and the peaks at δ = 6.51 ppm, 4.15-3.09, 2.07 ppm and 1.33 ppm are due to the pectin back-bone.

XRD studies of graft copolymers

Figure 5 shows the X-ray diffractograms of the pure pectin (A), PC-g-AMPS (B) and PC-g-SVBS (C). The pure pectin showed intense diffraction small peaks at 2θ = 23, 25, 26, 28, 30°. For the grafted pectin there are two diffraction peaks at the diffraction angles (2θ) 20° and 25°. The first one is crystalline nature, but after the grafting, the crystalline nature was disappeared due to the poly styrene sulphonate chains added on to the pectin backbone. As shown in Fig, in accordance with the characteristic diffraction peaks of Pectin, and the disappearance of sharp peaks after the grafting with SVBS, indicates that the loss of the crystalline nature and the amorphous behaviour in Pectin-g-SVBS is acquired due to SVBS [4,5].

DSC

Figure 8 shows the DSC thermograms of pure pectin (B), PC-g-AMPS (A) and PC-g-SVBS. From these thermograms, the endothermic peaks called decomposition curves. For pectin which shows a characteristic melting curve at 180 ^{o}C, and a decomposition curve a 450 ^{o}C. Thermogram is belongs to PC-g-AMPS, which shows three endothermic curves at 150, 330, 350 ^{o}C, out of which the peak at 150 ^{o}C is melting curve and remaining are various decomposition curves of polyAMPS. The thermogram belongs to PC-g-SVBS, the firs peak at 85 ^{o}C may be the melting curve of grafted polymer which is merged with water content (so its intensity is high compared to curve B) and the peak 460 ^{o}C is the decomposition of poly SVBS [6,7,].

References:

[1]. Prasad, S. S., Rao, K. M., Reddy, P. R. S., Reddy, N. S., Rao, K. K., & Subha, M. C. S. (2012). Synthesis and characterisation of guar gum-g-poly (acrylamidoglycolic acid) by redox initiator. Indian J Adv Chem Sci, 1(1), 28-32.

[2]. Anirudhan, T. S., & Rejeena, S. R. (2014). Poly (acrylic acid-co-acrylamide-co-2-acrylamido-2-methyl-1-propanesulfonic acid)-grafted nanocellulose/poly (vinyl alcohol) composite for the in vitro gastrointestinal release of amoxicillin. Journal of Applied Polymer Science, 131(17).

[3]. Willson, T. R., Hamerton, I., Varcoe, J. R., & Bance-Soualhi, R. (2019). Radiation-grafted cation-exchange membranes: an initial ex situ feasibility study into their potential use in reverse electrodialysis. Sustainable Energy & Fuels, 3(7), 1682-1692.

[4]. Sui, C., Preece, J. A., & Zhang, Z. (2017). Novel polystyrene sulfonate–silica microspheres as a carrier of a water soluble inorganic salt (KCl) for its sustained release, via a dual-release mechanism. RSC advances, 7(1), 478-481.

[5]. Jayakumar, G. C., Usharani, N., Kawakami, K., Rao, J. R., & Nair, B. U. (2014). Studies on the physico-chemical characteristics of collagen–pectin composites. RSC Advances, 4(109), 63840-63849.

[6]. Pereira, L. M., Carmello-Guerreiro, S. M., & Hubinger, M. D. (2009). Microscopic features, mechanical and thermal properties of osmotically dehydrated guavas. LWT-Food Science and Technology, 42(1), 378-384.

[7]. Martins, C. R., Ruggeri, G., & De Paoli, M. A. (2003). Synthesis in pilot plant scale and physical properties of sulfonated polystyrene. Journal of the Brazilian Chemical Society, 14, 797-802.

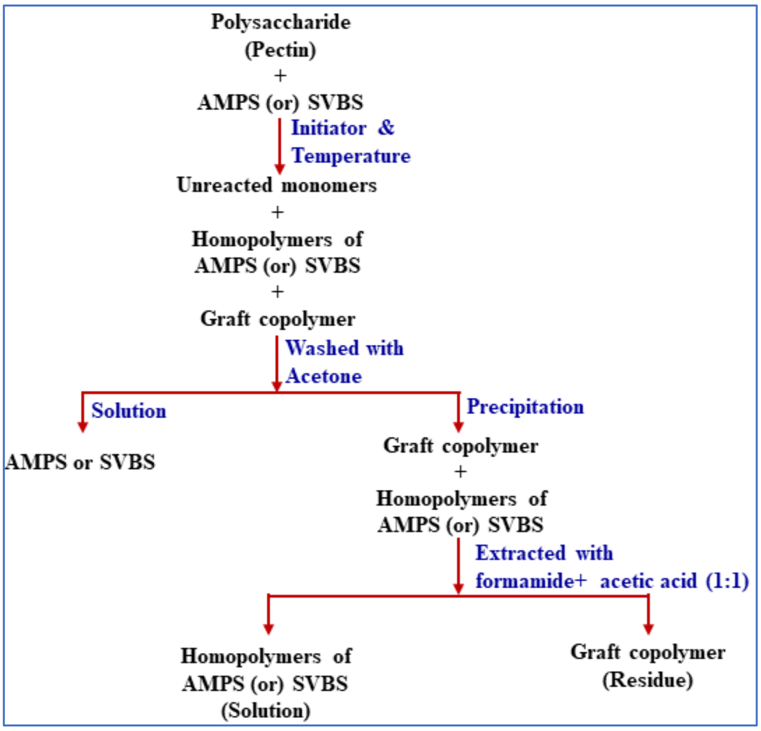

Scheme S1: Pectin graft copolymers preparation and purification.

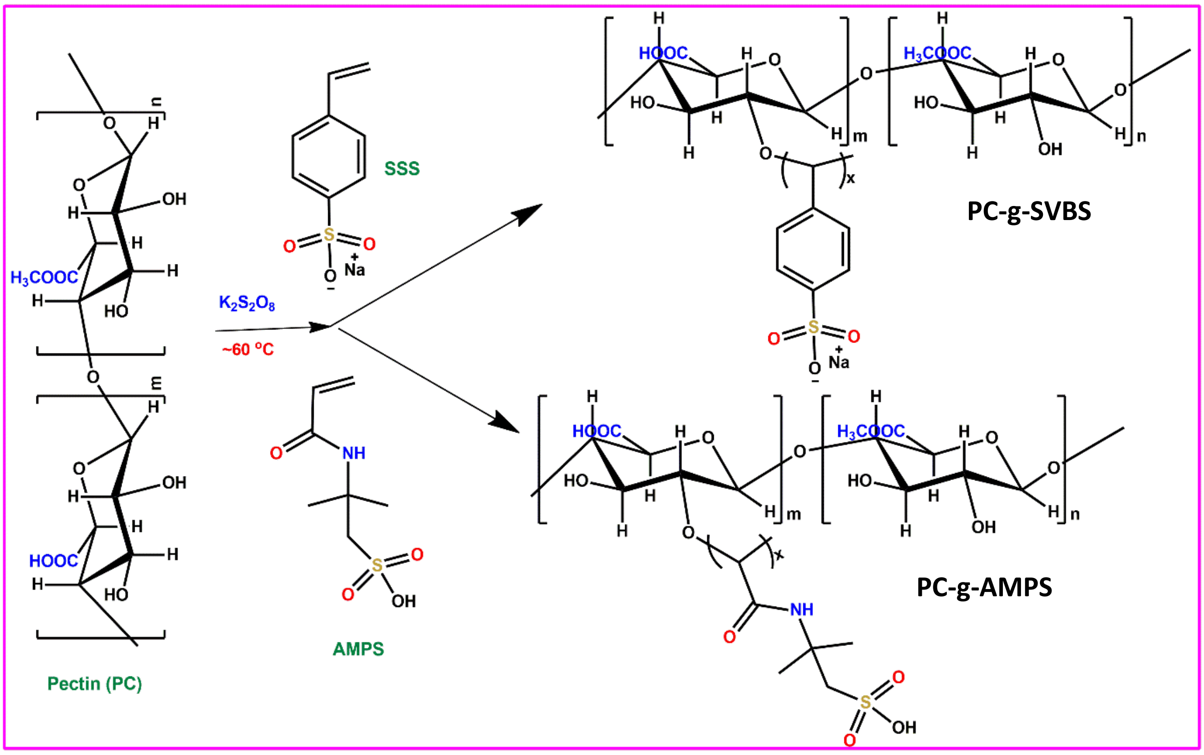

Scheme S2: Plasible schemactic chemistry of graft copolymerisation of AMPS and SVBS on to SA.

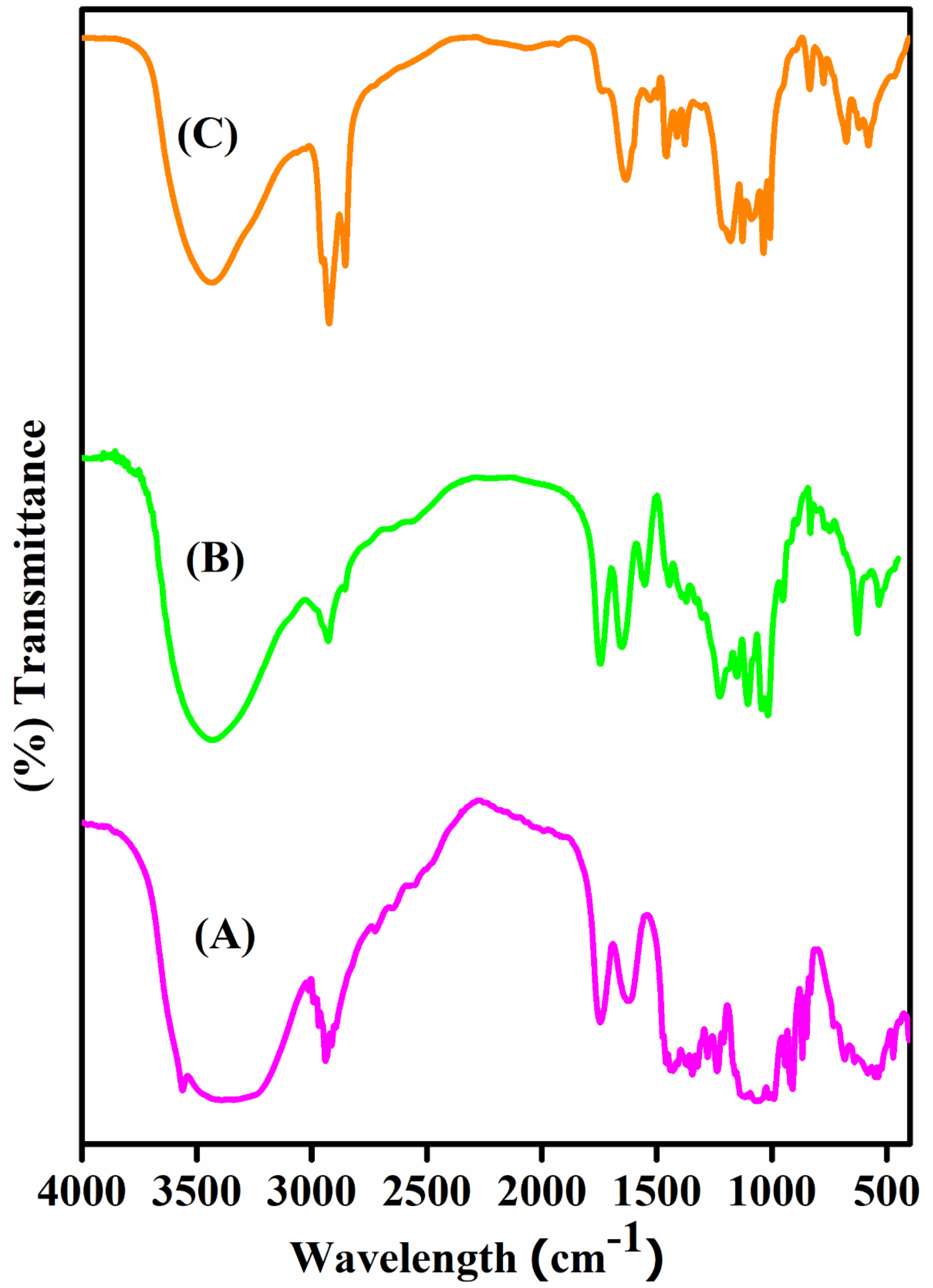

Figure S 1: FTIR spectrum of Pure pectin (A), pectin-g-AMPS (B) and pectin-g-SVBS (C).

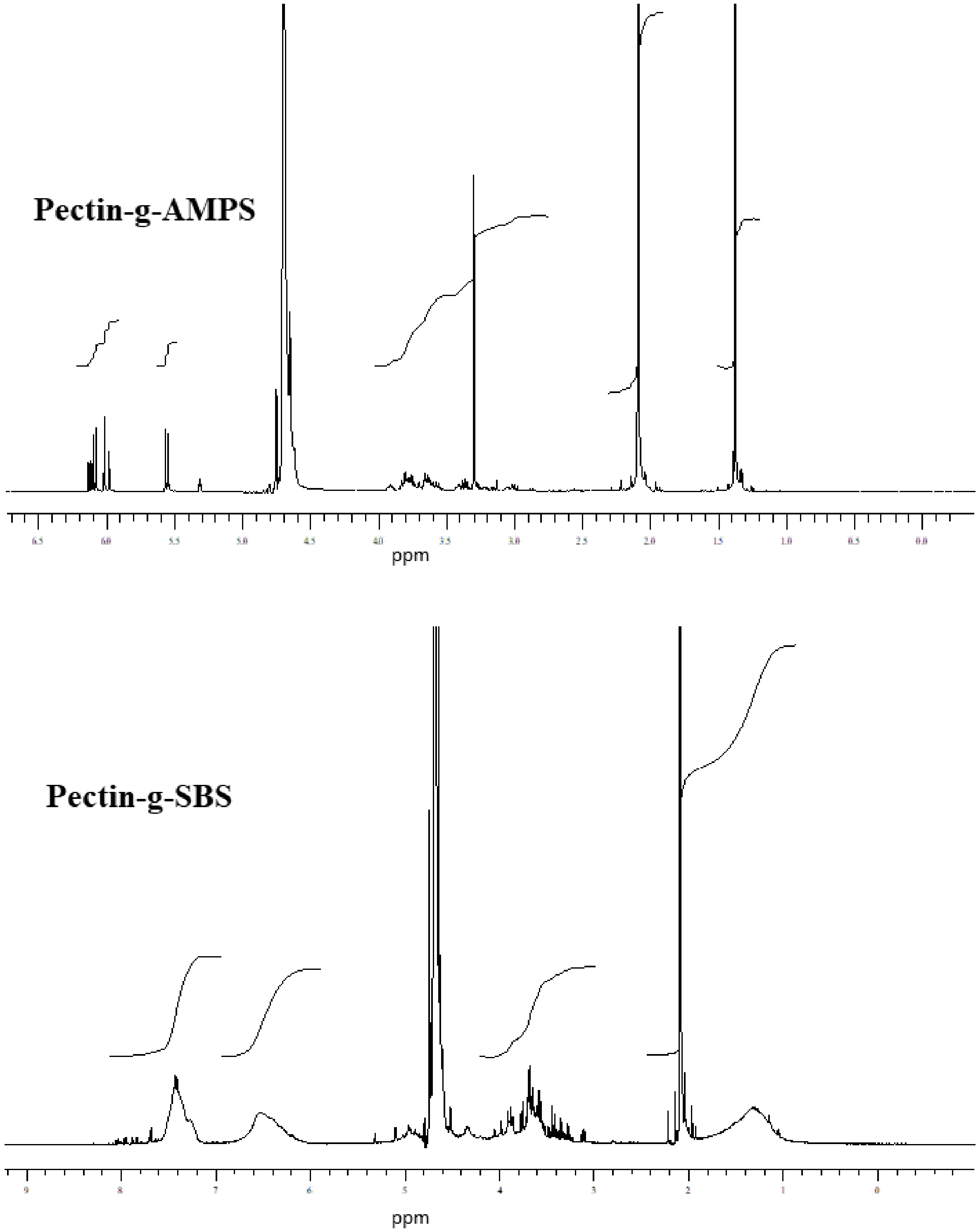

Figure S 2: NMR spectrum of pectin-g-AMPS and pectin-g-SVBS.

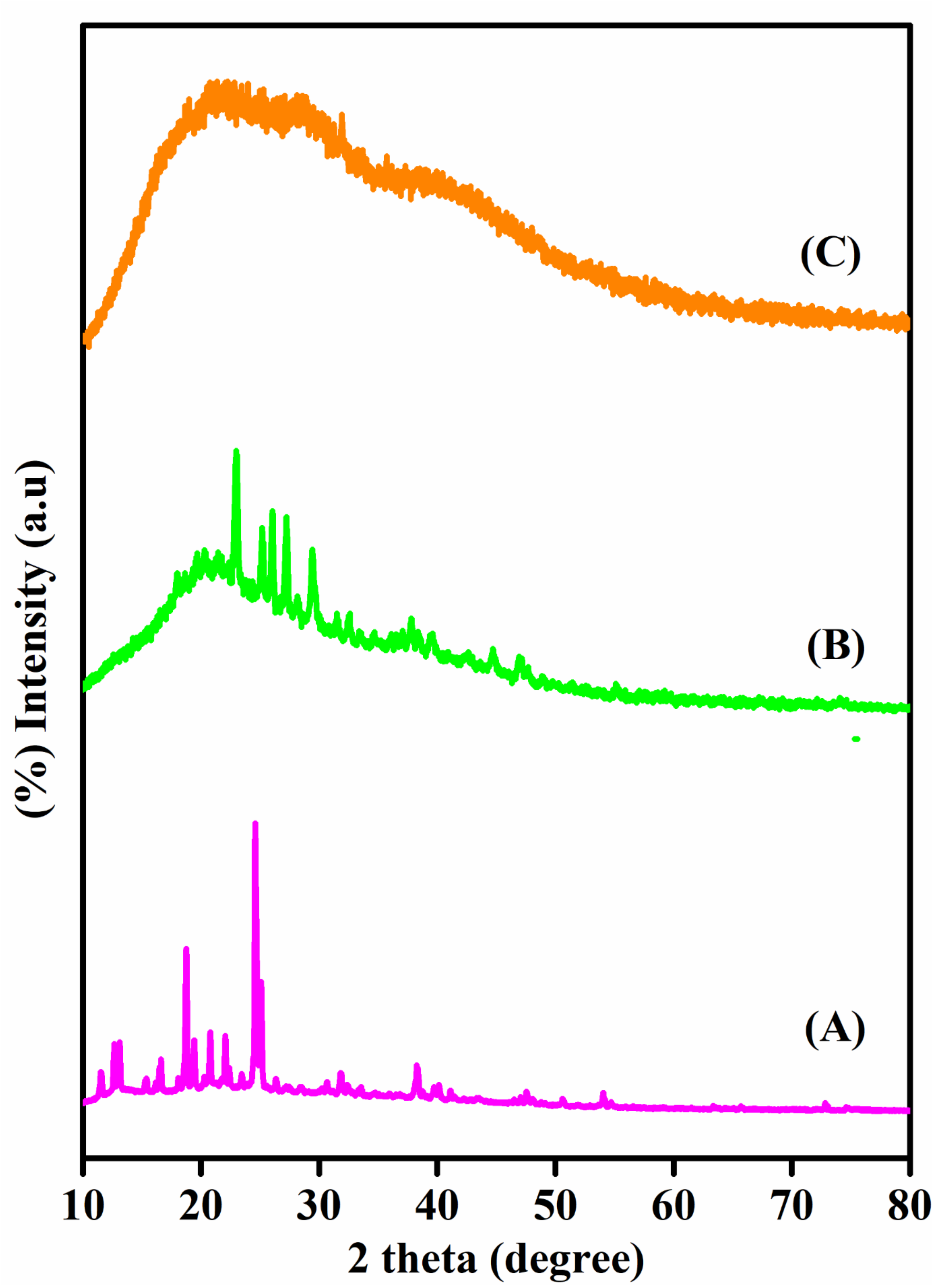

Figure S 3: XRD patterns of Pure pectin (A), pectin-g-AMPS(B) and pectin-g-SVBS (C).

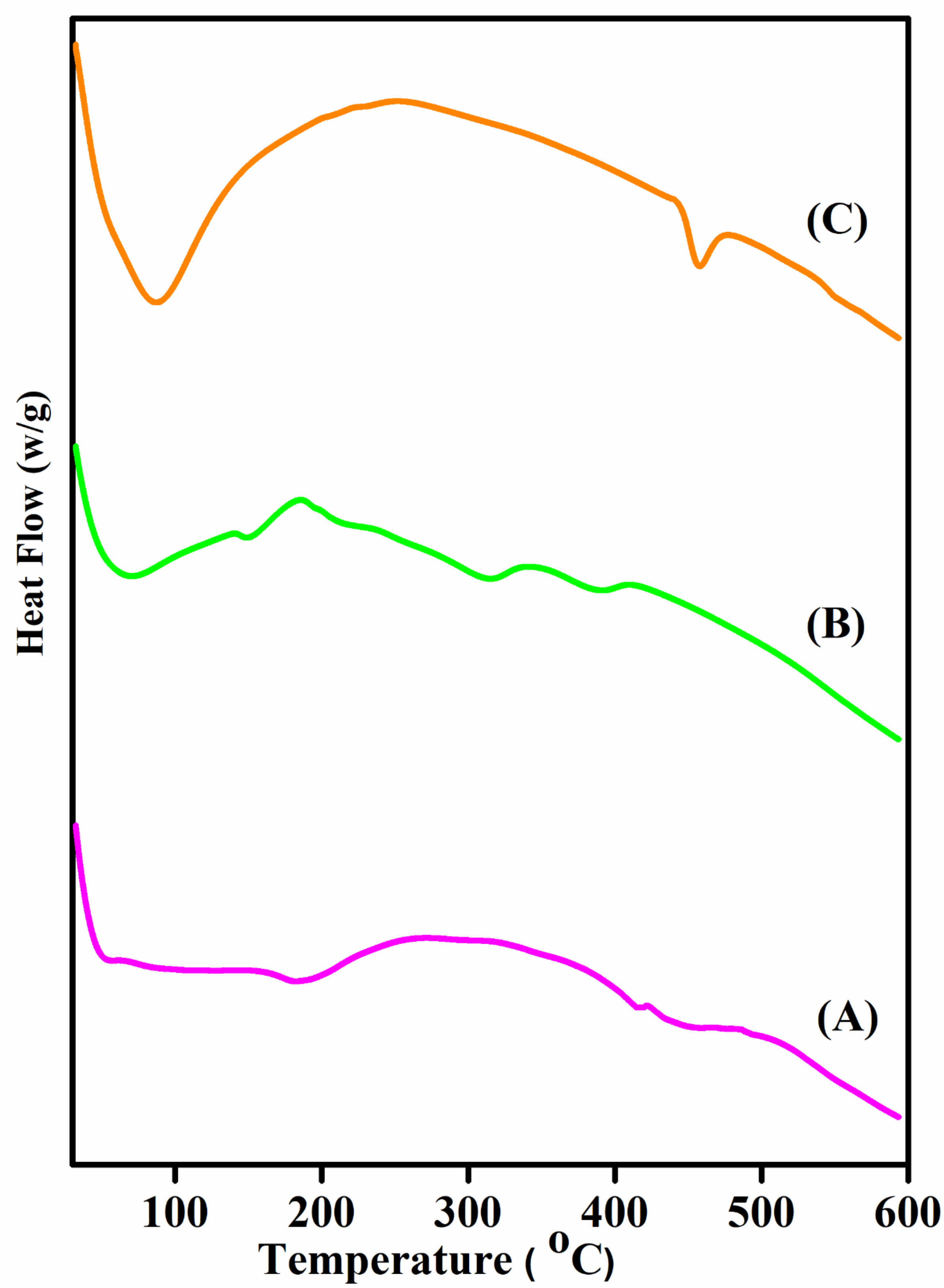

Figure S 4: DSC thermograms of pectin (B), pectin-g-AMPS (A) and pectin-g-SVBS (C).

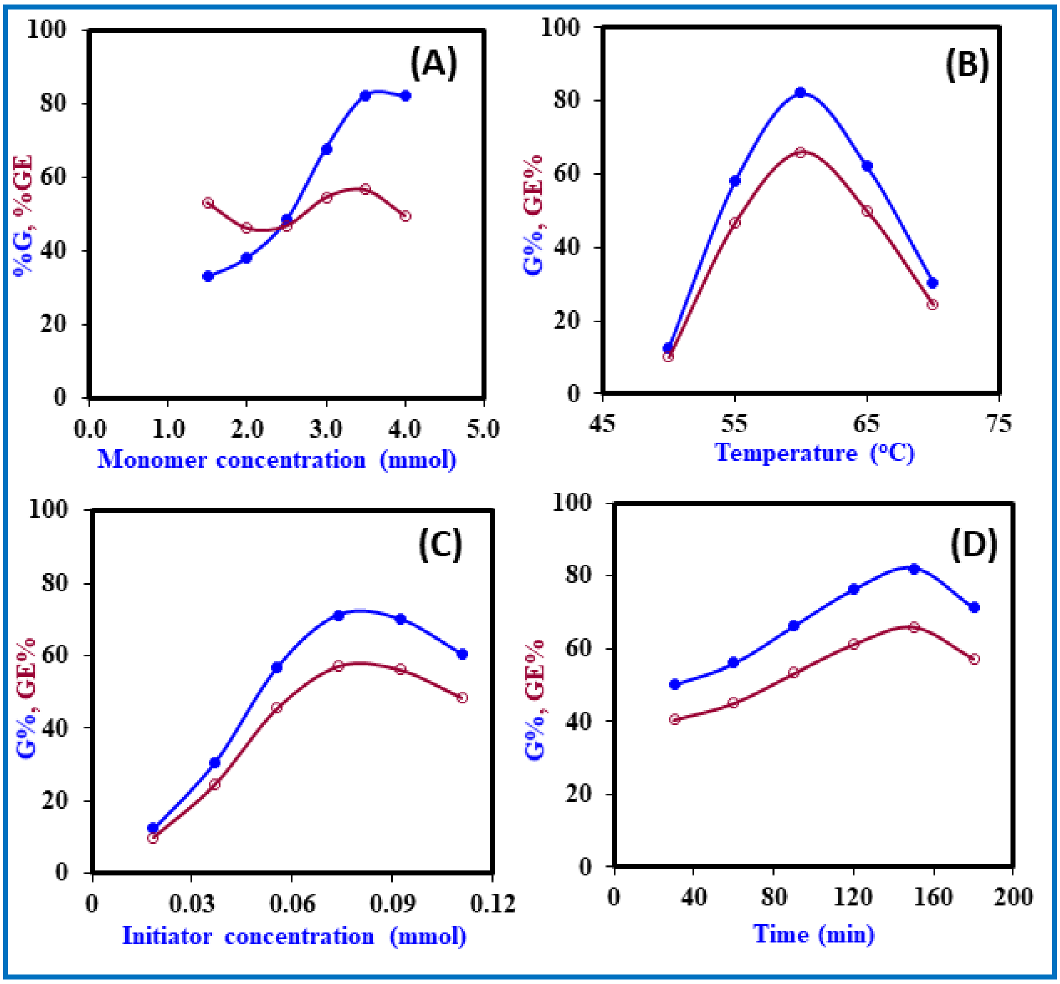

Figure S5: Effect of various reaction conditions on the grafting of AMPS onto the PC, prepared by KPS as initiator. **(A)** Effect of monomer concentration [Other reaction conditions: 0.5 g of PC; temperature: 60 °C; time: 150 min; initiator concentration 0.07 mol.]. **(B)** Effect of reaction temperature [Other reaction conditions: 0.5 g of PC; Monomer concentration: 3 mmol; time: 150 min; initiator concentration 0.07 mol.] **(C)** Effect of initiator concentration [Other reaction conditions: 0.5 g of PC; Monomer concentration: 3 mmol; time: 150 min; temperature: 60 °C.] **(D)** Effect of reaction time. [Other reaction conditions: 0.5 g of PC; Monomer concentration: 3 mmol; initiator concentration 0.07 mol; temperature: 60 °C]

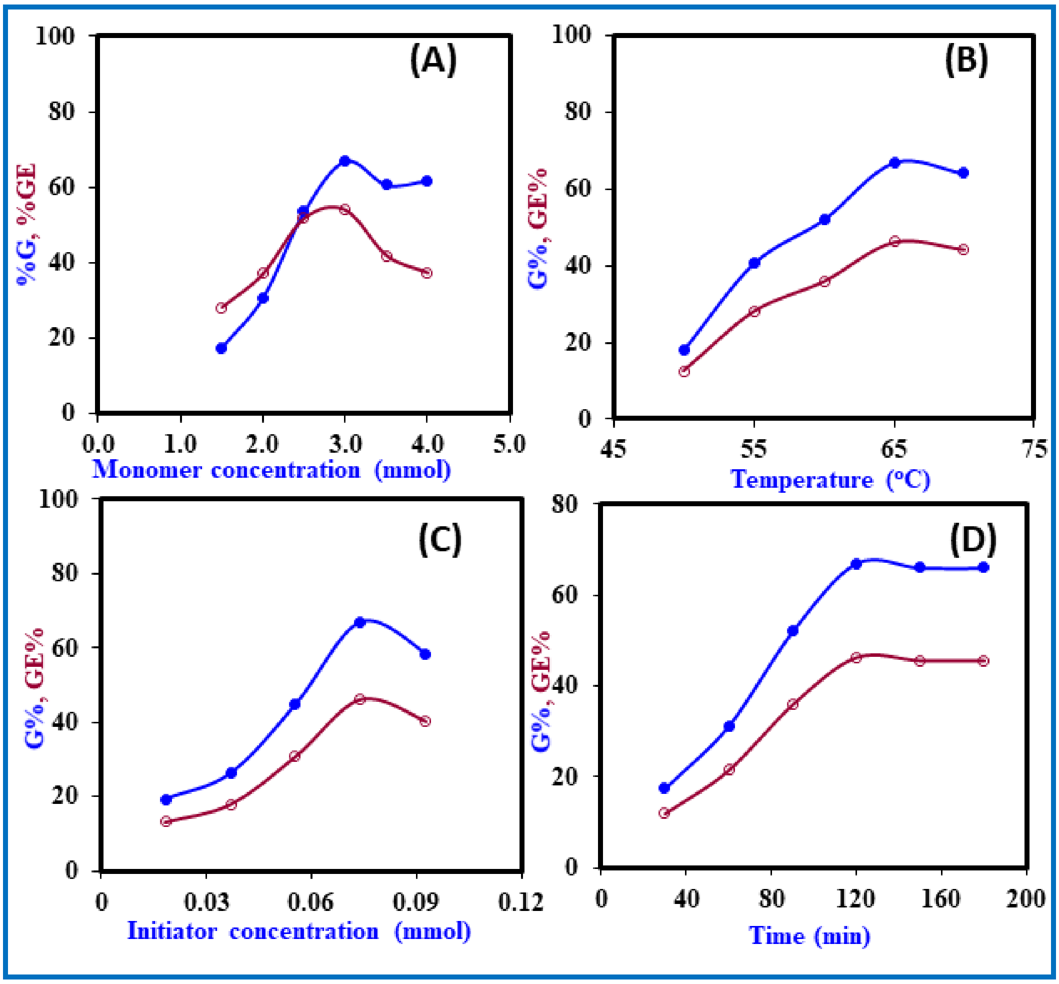

Figure S6: Effect of various reaction conditions on the grafting of SVBS onto the PC, prepared by KPS as initiator. **(A)** Effect of monomer (SVBS) concentration [Other reaction conditions: 0.5 g of PC; temperature: 65 °C; time: 120 min; initiator concentration 0.07 mmol.]. **(B)** Effect of reaction temperature [Other reaction conditions: 0.5 g of PC; Monomer concentration: 3 mmol; time: 120 min; initiator concentration 0.07 mmol.] **(C)** Effect of initiator concentration [Other reaction conditions: 0.5 g of PC; Monomer concentration: 3 mmol; time: 120 min; temperature: 65 °C.] **(D)** Effect of reaction time. [Other reaction conditions: 0.5 g of PC; Monomer concentration: 3 mmol; initiator concentration 0.07 mmol; temperature: 65 °C].

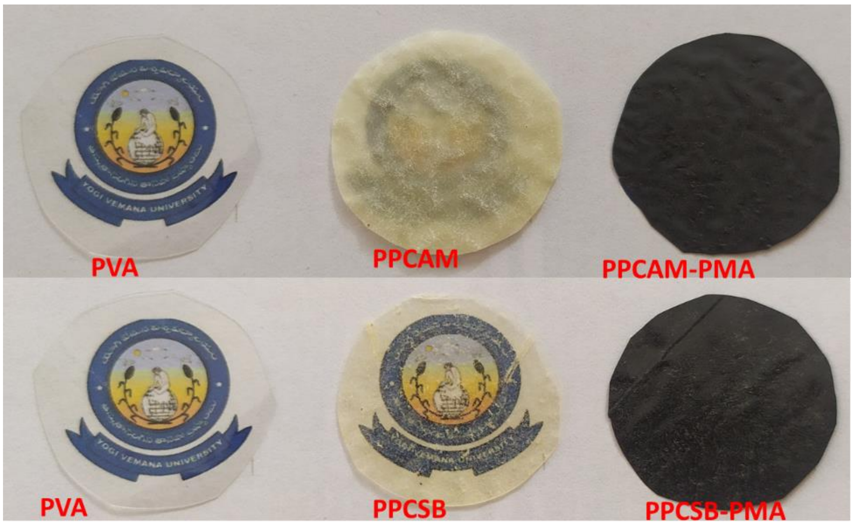

Figure S7: Digital Photographs of the various PEMs

Table S1: %Grafting, %grafting efficiency, %conversion, and %yield of PC-g-AMPS graft copolymer prepared by APS as initiator based on effect of various reaction conditions.

	**%Grafting**	**%Grafting Efficiency**	**%Conversion**	**%Yield**
**Temperature (°C)**	**Effect of temperature on grafting**			
50	12.64	10.16	90.58	50.21
55	58.14	46.76	127.17	70.49
60	82.16	66.07	146.49	81.19
65	62.06	49.91	130.33	72.24
70	30.40	24.45	104.87	58.12
**Time (min)**	**Effect of time on grafting**			
30	50.2	40.4	120.82	66.97
60	56.0	45.1	125.47	69.54
90	66.2	53.3	133.69	74.10
120	76.2	61.3	141.71	78.55
150	82.2	66.1	146.49	81.19
180	71.2	57.3	137.71	76.33
**KPS concentration (mmol)**	**Effect of initiator concentration on grafting**			
0.02	12.36	9.94	90.36	50.08
0.04	30.70	24.69	105.11	58.26
0.06	56.72	45.61	126.03	69.86
0.07	71.24	57.29	137.71	76.33
0.09	70.02	56.31	136.73	75.78
0.11	60.42	48.59	129.01	71.50
**AMPS concentration (mmol)**	**Effect of monomer concentration on grafting**			
1.5	33.01	53.09	213.93	82.02
2.0	38.18	46.06	166.68	75.55
2.5	48.38	46.69	143.19	72.87
3.0	67.73	54.47	134.89	74.76
3.5	82.04	56.55	125.48	74.28
4.0	82.02	49.47	109.78	68.48

Table S2: %Grafting, %grafting efficiency, %conversion, and %yield of PC-g-SVBS graft copolymer prepared by KPS as initiator based on effect of various reaction conditions.

	**%Grafting**	**%Grafting Efficiency**	**%Conversion**	**%Yield**
**Temperature (^{o}C)**	**Effect of temperature on grafting**			
50	17.98	12.39	81.33	48.14
55	40.64	28.02	96.95	57.39
60	52.14	35.94	104.88	62.08
65	67.03	46.21	115.15	68.16
70	64.24	44.29	113.22	67.02
**Time (min)**	**Effect of time on grafting**			
30	17.38	11.98	80.92	47.90
60	31.24	21.54	90.47	53.55
90	52.02	35.86	104.80	62.03
120	67.03	46.21	115.15	68.16
150	66.04	45.53	114.46	67.75
180	65.98	45.48	114.42	67.73
**KPS concentration (mmol)**	**Effect of initiator concentration on grafting**			
0.02	19.26	13.28	82.21	48.66
0.04	26.24	18.09	87.02	51.51
0.06	44.82	30.89	99.82	59.09
0.07	67.03	46.20	115.13	68.15
0.09	58.46	40.30	109.23	64.66
**SVBS concentration (mmol)**	**Effect of monomer concentration on grafting**			
1.5	17.46	28.08	188.92	72.43
2.0	30.72	37.06	157.68	71.47
2.5	53.60	51.72	148.23	75.43
3.0	67.03	53.90	134.32	74.45
3.5	60.64	41.80	110.73	65.55
4.0	61.80	37.27	97.59	60.87

Table S3: In-vitro drug release studies of various kinetics mod

Sample code	Zero order		First order		Higuchi		Hixson-Crowell		Korsmeyer- Peppas					
									pH-1.2			pH -7.4		
	R	K_0	***R***	K_1	***R***	K_h	***R***	K_{hc}	***R***	***n***	***k***	***R***	***n***	***k***
PVA	0.85	5.12	0.723	4.03	0.723	1.75	0.731	3.35	0.943	8.21	5.32	0.943	0.81	5.82
Pectin-g-SSS	0.971	0.85	0.980	0.75	0.980	0.32	0.981	1.15	0.968	0.25	0.17	0.998	0.40	0.26
Pectin-g-AMPS	0.984	0.98	0.973	2.06	0.973	0.89	0.986	1.82	0.986	0.29	0.37	0.924	0.64	3.12
